# Establishing a minimum passing score for the rating scale in simulation training for surfactant administration using the LISA procedure

**DOI:** 10.1186/s12909-025-06778-8

**Published:** 2025-02-26

**Authors:** Hélène Rostoker, Bernard Guillois, Amaya Caradec, Clément Chollat

**Affiliations:** 1 Sorbonne Université, Department of Neonatal Paediatrics, Trousseau Hospital, APHP, Paris, F-75012 France; 2 Medical School, Caen Normandie University, Caen, F-14000 France; 3 P2ULSE, APHP Center, Trousseau Hospital, Sorbonne University, Paris, France; 4 Université Paris Cité, Inserm, NeuroDiderot, Paris, F-75019 France

**Keywords:** Simulation, Neonatology, LISA, Rating scale, Minimum rating score

## Abstract

**Introduction:**

In a previous study, a rating scale for simulation training on surfactant administration using the LISA (Less Invasive Surfactant Administration) procedure was developed and validated. Our objective was to determine a minimum passing score for this rating scale to use it so that it could be used for normative and certifying evaluation.

**Methods:**

The LISA scale comprises 8 categories and 25 items. It was developed and agreed upon by a panel of 12 LISA procedure experts, and subsequently validated through simulation sessions involving 40 learners. Two independent assessors evaluated these 40 simulations. The Cronbach’s alpha score for this scale is 0.72, the R-squared value is 0.99, and the intra-class correlation coefficient is 0.92. Three different methods were employed to establish the minimum passing score: the Angoff method, the Borderline group method, and the Contrasting Group method. For the Angoff method, we enlisted 5 experts from the panel who developed the rating scale. In the Borderline Group method, the two assessors evaluated the 40 simulations following the prescribed methodology. For the Contrasting Group method, the outcomes of various simulation sessions were analyzed.

**Results:**

Using the Angoff method, the minimum passing score was determined to be 35 out of 50, equivalent to 70.6% (standard deviation: 15.8%). Employing the Borderline Group method yielded a minimum passing score of 31.70 out of 50, i.e., 63.4%. Finally, utilizing the Contrasting Group method, the minimum passing score was found to be 33 out of 50, or 66%.

**Conclusion:**

The three methods employed resulted in varying minimum passing scores. A higher score is likely to ensure enhanced safety and quality of patient care, while also facilitating the learner’s progression in simulator training. We recommend considering a minimum passing score of 35 out of 50.

## Introduction

The Less Invasive Surfactant Administration (LISA) procedure, which involves using a thin catheter to deliver surfactant while maintaining spontaneous breathing on CPAP [1], reduces the need for mechanical ventilation, the combined risk of death or bronchopulmonary dysplasia, and intraventricular hemorrhage compared to the traditional procedure (intubation and surfactant administration through an endotracheal tube) [2–4]. This procedure have emerged as the standard of care for surfactant administration and become widely adopted in neonatal units [5–7]. The teaching of LISA through simulation is still in development, with formal validation of this skill pending. In our prior study, the EVALISA study, a rating scale for teaching the LISA procedure in simulation training was developed using the Downing method and approved by a panel of LISA experts following the Delphi method [8]. It exhibits satisfactory validity, with a Cronbach’s alpha score of 0.72, and good reproducibility, with an intra-class correlation coefficient of 0.91 and a linear coefficient of determination of 0.99. Establishing minimum passing scores is essential for the development of assessment scales and examinations leading to certification of competency. While it is encouraged to integrate the standardization of minimum passing scores into the design of the assessment scales, few studies have adopted this approach [9, 10]. In this study, our objective is to establish a minimum passing score for the rating scale in simulation training of surfactant administration using the LISA procedure, employing three different validated methods.

## Materials and methods

### Institutional review board

This project obtained approval from the Research Ethics Committee of Sorbonne University, Paris, France (CER-2022-028). Informed consent was obtained from all subjects and participants signed a waiver of image rights.

### Methods for determination of minimum passing score

Rigorous methodologies outlined in the literature are utilized to establish minimum passing scores [11–14]. The minimum passing score can be defined either by an absolute standard or a relative standard. An absolute standard is defined by items assessed by independent judges, as in the Angoff method. A relative standard is defined by comparing the performance of one group against another, as seen in the Borderline Group or Contrasting Group methods.

### Angoff method

The Angoff method comprises three primary stages: [14]

- Judges delineate the characteristics of the “borderline student”.
- Each judge independently estimates the score that the “borderline student” would receive for each item, rating them between 0 and 100%.
- The score for each item and the total score are calculated by averaging the percentages assigned by each judge. Judges’ scores must align closely, with discrepancies of more than 30% requiring justification until consensus is reached.

In this study, experts involved in creating the rating scale were contacted via email. These experts are practitioners who regularly employ the LISA procedure in their professional roles and are engaged in student training. The objective was to enlist a minimum of 5 judges from those involved in developing the rating scale [15, 16]. 

### Borderline Group method

In this approach, judges closely monitor each participant’s performance. After observing several instances, each judge assigns an overall performance score to each participant: 1 = Fail, 2 = Borderline, 3 = Pass. Concurrently, each participant obtains a score using the rating scale. The minimum passing score is determined by averaging the scores of individuals in the Borderline group. The minimum passing score was determined by calculating the average score of students who received a rating of 2 (‘Borderline’) in the judges’ assessment.

### Contrasting Group method

This method also facilitates the determination of a minimum passing score. It involves two categories of students: “Experienced” students who are proficient in the assessed technique and “novice” students who lack experience with the technique. The minimum passing score is then established to effectively distinguish between “novice” and “experienced” students. Judges evaluate the performance of both “novice” and “experienced” student using the EVALISA rating scale. The distribution of scores for both groups is then plotted, and the minimal passing score is identified at the intersection of these score distributions. In EVALISA study, “Experienced” students in the LISA procedure had completed more than 5 LISAs in their careers. Their performances were assessed by two independent judges. “Novices” in the LISA procedure had completed fewer than 5 LISAs in their careers and were evaluated by the same judges. Overall scores are taken from the initial EVALISA study [8]. 

### Judges recruitment

For the Angoff method, 5 judges were recruited from the experts panel which created the EVALISA scale. Two independent judges (HR and BG) were recruited to determine the minimum passing scores using the Borderline Group and Contrasting Group methods. HR and BG, both experienced neonatologists, were selected for their expertise in the LISA procedure, their direct involvement in developing and validating the rating scale, and their extensive experience with simulation-based training, demonstrating a strong understanding of the scale’s content and application. While it is usual to employ 5 or more judges, only 2 were employed due to logistical constraints [13]. Despite the limited number, the reliability of their evaluations was ensured by high inter-rater agreement, as evidenced by an intra-class correlation coefficient (ICC) of 0.91 [8]. Additionally, both judges underwent thorough training and calibration sessions to minimize variability in their assessments.

### Statistical analysis

Results are presented in average with standard deviation for the Angoff and the Borderline Group methods. Two optimal curves were generated using the mean scores and standard deviations of the novice and experienced groups, with the pass-fail threshold established at the point where the curves intersected, following the contrasting groups standard-setting method [11]. The statistical analysis was carried out on Excel^®^ version 2205, published by Microsoft®.

## Results

### Angoff method

The 12 experts who participated in developing the rating scale were contacted, and 5 agreed to participate in this study. These 5 experts established a minimum score out of 100 for each item. The standard deviation for each item was found to be less than 30%. The minimum passing score, determined by averaging the scores provided for each item by the 5 judges, was 70.6% (standard deviation: 15.8). Table 1 presents the average scores per item as well as the standard deviation of the scores assigned by the assessors.

**Table 1:**
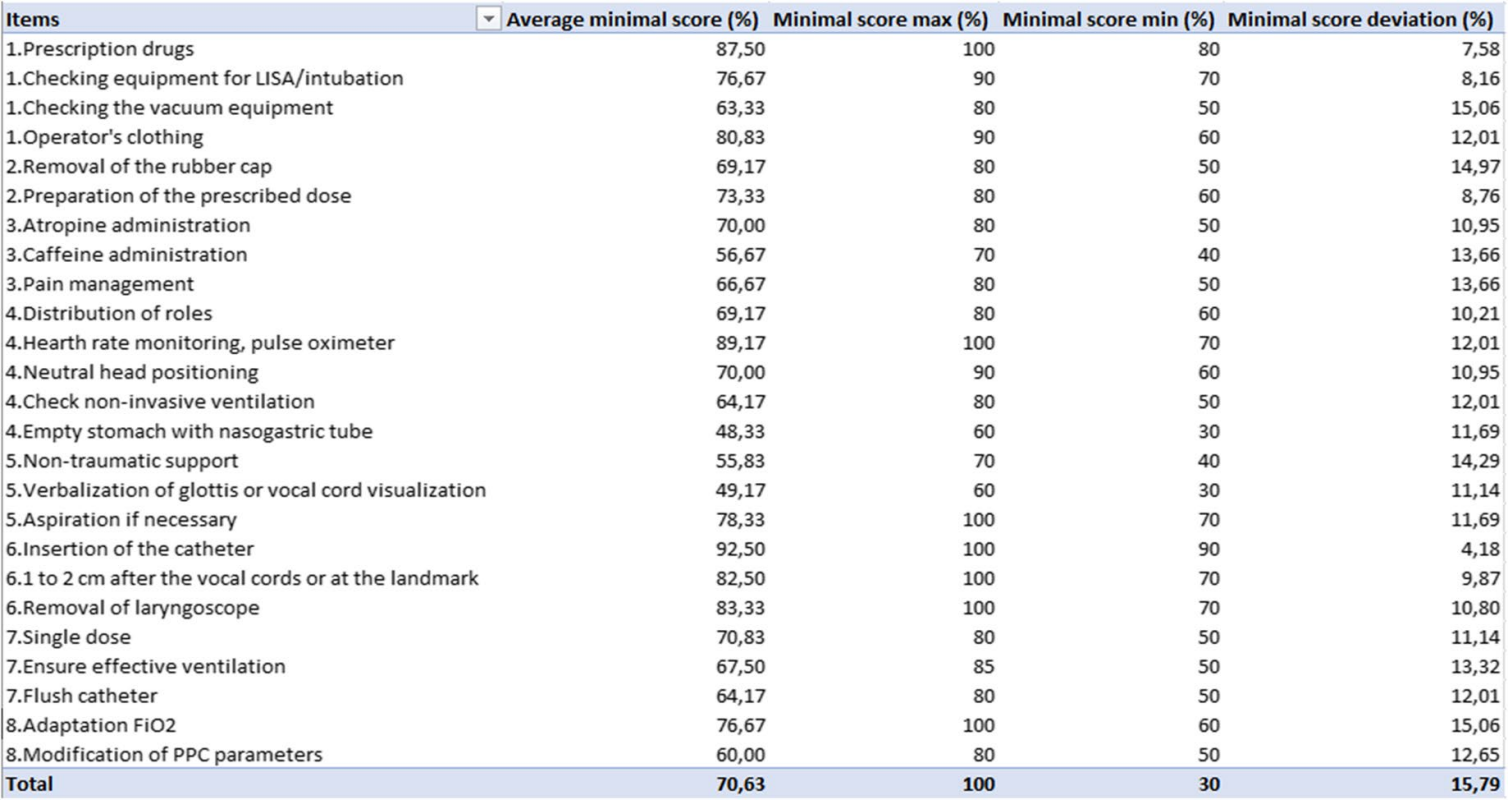
Angoff method results

### Borderline Group method

The two independent judges (HR and BG) who evaluated the performances after the simulations agreed to re-evaluate them using the Borderline Group method. Ultimately, 13 performances were classified as belonging to the “Borderline” group. The average score for the performances in this group was 31.70 out of 50, equivalent to 63.4%. These findings are detailed in Table 2.

**Table 2:** Bordeline Group results

Groups	Average of Notes (*n*)	Min. of Notes (*n*)	Max. of Notes (*n*)	StdDev of Notes (*n*)	Number of passages (*n*)
FAIL	25,57	15	31	4,26	7
BORDERLINE	29,42	22	35	3,69	13
PASS	37,75	32	44	2,83	12
*Not in the same group after the judge’s evaluation*					8
**Total**	**31**,**70**	**15**	**44**	**6**,**04**	**40**

### Contrasting Group method

**Fig. 1:**
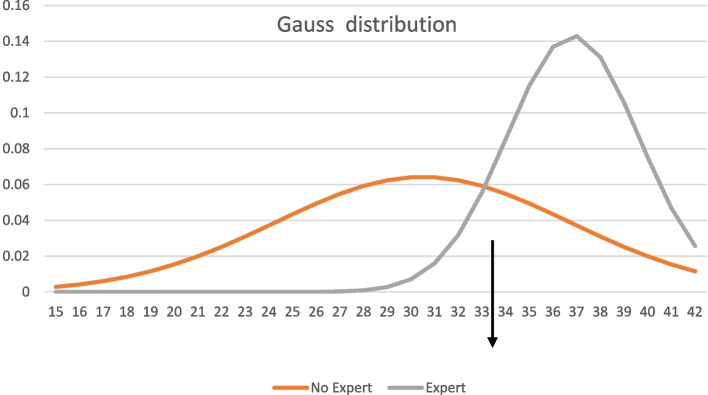
Contrasting Group results

During the simulation sessions held in February 2022, we identified 6 experts in the LISA procedure, characterized by having conducted more than 5 LISAs in their careers, alongside 34 novices, with fewer than 5 LISAs performed. The scores in each category were distributed in a Gaussian manner. Notably, the two Gaussian curves intersected precisely at 33 out of 50, corresponding to 66%. These intersecting Gaussian curves are shown in Fig. 1.

**Fig. 2:**
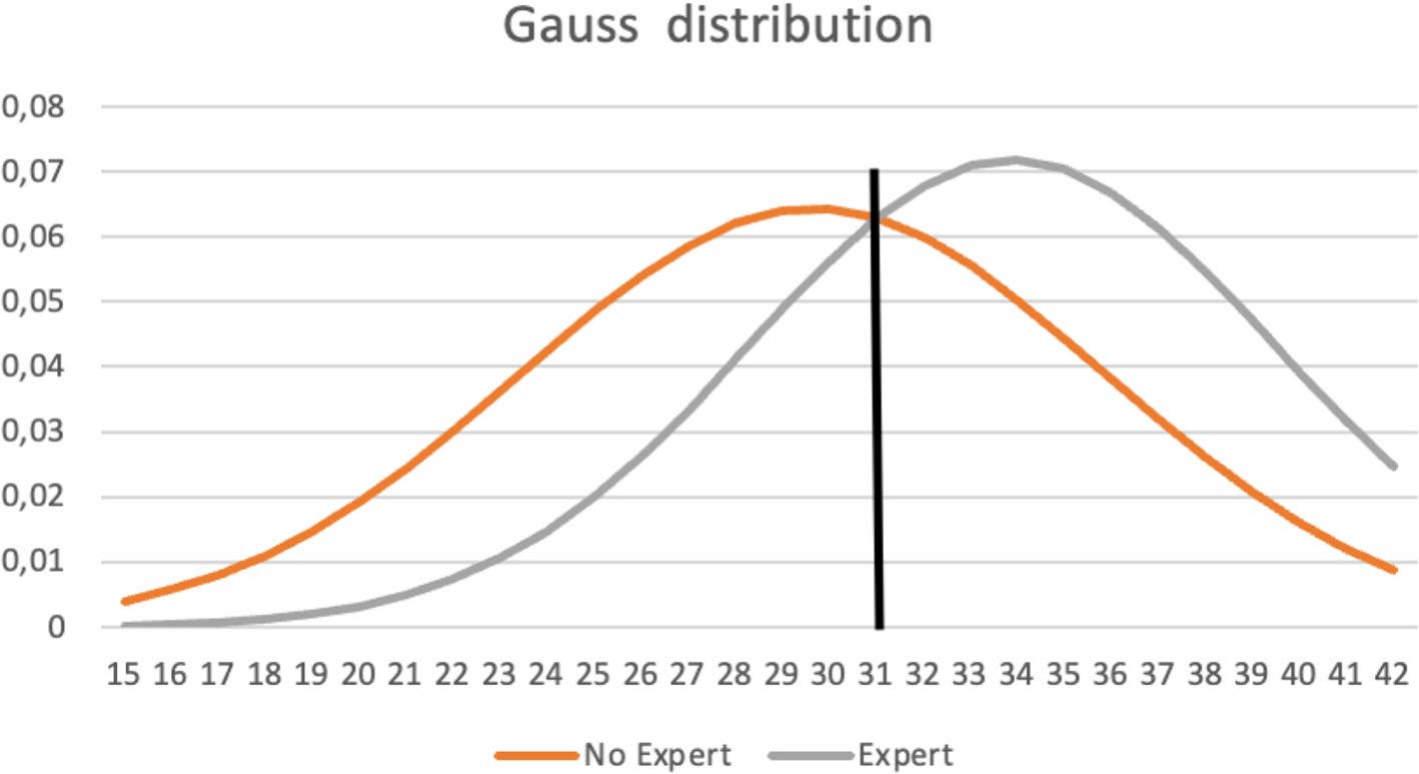
Contrasting group (intubation experts)

Seventeen learners had conducted over 10 intubations throughout their careers. Intubation and LISA procedures share several commonalities, including environmental checks, sedation administration, and visualization of vocal cords. Recognizing these similarities, we opted to apply the Contrasting Group method to the cohort of intubation experts as well. We classified learners as experts if they had performed more than 10 intubations in their professional careers. The distribution of scores within each category followed a Gaussian pattern. Notably, the two Gaussian curves intersected at 31 out of 50, or 62%. The intersecting Gaussian curves depicting intubation experts are shown in Fig. 2.

## Discussion

Through the application of these three distinct methods-the Angoff, Borederline group and Contrasting Group methods-this study has established a range of minimum passing score varying from 63.4 to 70.6%. Each method offers unique strengths and limitations, allowing for a comprehensive exploration of standard-seting apprpoaches in this context.

The Angoff method, a tried-and-tested classical approach, has been widely utilized for developing minimum passing scores [17]. One of its key strengths lies in its detailed description in the literature and its adaptability to performance test development [13, 14]. This method offers reliability as judges independently assess each item on the rating scale, thereby ensuring the overall score’s accountability [18]. However, Verheggen et al. [19] showed that individual judgement of the item not only reflects its difficulty but also the judge’s inherent rigor and disciplinary expertise. Hence careful selection of judges is paramount, ensuring they can accurately portray the “borderline” student and possess adequate proficiency to address all assessments tool queries. In our study, judges not only contributed to the rating scale’s development, but also exhibited competence and training in the LISA procedure. Furthermore, the definition of the “borderline” student was established through discussions between the study designer and the judges.

The Borderline Group method offers the advantage of focusing on the candidate rather than individual items on the rating scale [18]. Consequently, it closely aligns with learner’s actual skill levels [20]. Moreover, it is straightforward to implement post-simulation sessions, requiring minimal additional time for judges to categorize performances according to the three Borderline method classifications. To employ this method effectively, it is imperative that the judges possess qualifications in the taught method and are accustomed to evaluating simulator performances [18]. In this study, both judges conducted numerous assessments of simulation sessions and were well-versed in the LISA procedure. Notably, there were no statistically significant differences between the scores assigned by the two observers. Furthermore, familiarity with the content of the rating scale is crucial for both judges. In this instance, both judges were involved in developing the tool and executing assessments using it, thereby demonstrating a strong command of the content.

The Borderline Group method calculates the minimum passing score by averaging the scores of participants in the “borderline” group. However, this calculation is inevitably influenced by the lowest scores, which tend to lower the average. In our study, this method yielded the lowest minimum score, possibly due to its susceptibility to the lowest scores among learners in the “Borderline” group.

The Contrasting Group method is that it focuses on candidates who are already proficient in the technique, allowing the expected level of the participant to be determined based on the actual level of proficiency of those who perform the technique on a regular basis [21, 22]. This method mitigates potential bias from judges evaluating items they have not performed in the simulation. However, the effectiveness of the Contrasting Group method relies on having an adequate number of qualified learners who are experts in the method to achieve a reliable Gaussian curve. In the cohort of learners who participated in the simulation sessions, there were 6 individuals identified as experienced in the LISA procedure, as per the previously established criteria. Additionally, there were a total of 17 learners (42% of the population) who had conducted more than 10 intubations throughout their professional careers. Given the similarities between these two procedures, including exposure to the laryngoscope, locating the vocal cords, and managing analgesia, one might have considered defining expertise based on experience with more than 10 intubations. However, such an approach could have potentially lowered the minimal passing score and compromised the accuracy of the study in terms of evaluating the LISA procedure, which was our primary focus. Consequently, we opted to exclusively assess the minimum passing score using the Contrasting Group method on experts in the LISA procedure, despite the smaller sample size. Nevertheless, the convergence of the two Gaussian curves can be attributed to the significant number of individuals experienced in intubation who were not classified as LISA procedure experts. These individuals demonstrated superior performance compared to novices in both intubation and the LISA procedure, contributing to the observed trend.

### Strengths and limitations

One notable strength of this study is its rigorous methodology, involving the use of three complementary methods to determine the minimum passing score. This multi-method approach enhances the reliability and applicability of the findings by providing convergent evidence across different standard-setting techniques. However, a significant limitation lies in the reliance on only two judges for the Borderline Group and Contrasting Group methods. Although these judges possessed significant qualifications, extensive expertise in the LISA procedure, and were directly involved in creating the rating scale, the limited number of evaluators may have introduced bias and reduced the generalizability of the findings. The decision to use only two raters was influenced by logistical constraints. However, the high level of agreement between the two raters in the initial study mitigates this limitation to some extent. As a reminder, we highlighted the excellent interrater reliability of the EVALISA rating scale (*R*^2^ = 0.99 and ICC = 0.91) [8].

### Implications for practice

The findings of this study have significant implications for the training and evaluation of healthcare professionals in simulation-based education. The consistent range of minimum passing scores derived from the three methods provides educators with evidence-based benchmarks for assessing competency in the LISA procedure. These thresholds ensure that learners achieve a high level of proficiency, thereby promoting patient safety and the quality of care. The minimum passing scores identified in this study consistently fall within a comparable range, from 63 to 70.6%. In mastery-based education, learners progress only after demonstrating proficiency, and performance is prioritized over fixed training durations. This ensures that all learners, regardless of the time required, achieve a consistent standard of competency, which is critical for patient safety and effective clinical practice [23]. However, as mastery learning highlights, the focus should not solely be on achieving a minimal standard but on preparing learners to succeed in subsequent stages of training and practice. Integrating specific critical elements, such as those related to patient safety, into the rating scale, as is the case in the EVALISA scale, could further enhance its impact. Although our study did not explicitly assess items related to patient safety, many items in the rating scale emphasize preparation and anticipation, which could contribute to safer procedures and environments [24]. 

## Conclusion

In conclusion, we have established three minimum success scores for the LISA scale in surfactant administration simulation training, ranging from 63.4 to 70.6%. A higher score not only enhances patient safety but also prompts additional simulation training in the event of failure. We recommend considering a minimum passing score of 35 out of 50.

## Data Availability

The datasets used and analyzed during the current study are available from the corresponding author on reasonable request.

## Abbreviations

LISA: Less Invasive Surfactant Administration
P2ULSE: Plateforme Pluridisciplinaire Hospitalo-Universitaire de e-Learning et de Simulation de l'Est Parisien
